# A Biofeedback-Based Mobile App With Serious Games for Young Adults With Anxiety in the United Arab Emirates: Development and Usability Study

**DOI:** 10.2196/36936

**Published:** 2022-08-02

**Authors:** Mariam Almeqbaali, Sofia Ouhbi, Mohamed Adel Serhani, Leena Amiri, Reem K Jan, Nazar Zaki, Ayman Sharaf, Abdulla Al Helali, Eisa Almheiri

**Affiliations:** 1 Department of Computer Science and Software Engineering College of Information Technology United Arab Emirates University Al Ain, Abu Dhabi United Arab Emirates; 2 Department of Information Systems and Security College of Information Technology United Arab Emirates University Al Ain, Abu Dhabi United Arab Emirates; 3 Department of Psychiatry College of Medicine and Health Sciences United Arab Emirates University Al Ain, Abu Dhabi United Arab Emirates; 4 College of Medicine Mohammed Bin Rashid University of Medicine and Health Sciences Dubai United Arab Emirates

**Keywords:** connected mental health, mental health, anxiety, digital game, biofeedback, app, serious game, gaming, gamification, young adult, user-centered design, stress, stress relief, user-centred design, youth, user feedback, user experience, usability, user need, development, mHealth, mobile health

## Abstract

**Background:**

Following the outbreak of COVID-19, several studies have reported that young adults encountered a rise in anxiety symptoms, which could negatively affect their quality of life. Promising evidence suggests that mobile apps with biofeedback, serious games, breathing exercises, and positive messaging, among other features, are useful for anxiety self-management and treatment.

**Objective:**

This study aimed to develop and evaluate the usability of a biofeedback-based app with serious games for young adults with anxiety in the United Arab Emirates (UAE).

**Methods:**

This study consists of two phases: Phase I describes the design and development of the app, while Phase II presents the results of a usability evaluation by experts. To elicit the app’s requirements during Phase I, we conducted (1) a survey to investigate preferences of young adults in the UAE for mobile games for stress relief; (2) an analysis of serious games for anxiety; and (3) interviews with mental health professionals and young adults in the UAE. In Phase II, five experts tested the usability of the developed app using a set of Nielsen’s usability heuristics.

**Results:**

A fully functional biofeedback-based app with serious games was co-designed with mental health professionals. The app included 4 games (ie, a biofeedback game, card game, arcade game, and memory game), 2 relaxation techniques (ie, a breathing exercise and yoga videos), and 2 additional features (ie, positive messaging and a mood tracking calendar). The results of Phase II showed that the developed app is efficient, simple, and easy to use. Overall, the app design scored an average of 4 out of 5.

**Conclusions:**

The elicitation techniques used in Phase I resulted in the development of an easy-to-use app for the self-management of anxiety. Further research is required to determine the app’s usability and effectiveness in the target population.

## Introduction

Due to the COVID-19 pandemic and the preventive measures imposed to limit the spread of the virus, such as lockdowns and social distancing, young adults in the United Arab Emirates (UAE) have reported increased stress from work, home, and finances [1], which potentially puts them at risk for psychological issues including anxiety [2,3]. Before COVID-19, anxiety affected 20%-25% of adult patients in the UAE, who are considered a psychologically vulnerable group. The prevalence of anxiety grew following the emergence of COVID-19, and it now affects 55.7% of the adult population in the UAE [4]. Anxiety constitutes a normal response to stressful events; however, when it is persistent, excessive, and left untreated, it can lead to serious problems [5]. Anxiety has been recognized to negatively impact the quality of life and psychosocial functioning [6], and to be a predictor of a wide range of mental disorders [7]. Given the negative impact of anxiety on health, as well as the medical and financial burdens of mental health services, an effective method for managing anxiety in young adults is required.

One proposed solution is connected mental health, which is the use of information and communication technology in supporting mental health care. Due to the availability and popularity of mobile devices [8], mobile apps are one of the most popular connected mental health approaches, with many people reporting using their mobile devices to access health-related information and expressing an interest in using apps to track their anxiety [9,10]. A recent systematic review of highly rated anxiety apps has identified several apps that people with anxiety can use independently to practice relaxation and management methods [11]. Breathing exercises, yoga, and motivational quotes were among these management methods. Furthermore, half of these apps used gamification features to keep users engaged.

Studies have identified that games, especially commercial video games, have a significant impact on cognitive function, specifically on attentional control [12], and mental health including anxiety and stress [13]. In addition, games that are specifically designed to address a problem or teach a certain ability have had a lot of success [14]. Serious games are games with a main purpose other than pure entertainment, such as games used for education and health care purposes, with the added value of entertainment and competition. Researchers have demonstrated that serious games are comparable to traditional therapies, and for some users, can be more fun and acceptable [15]. Serious games also facilitate enhanced user engagement and increased motivation, which ultimately can improve treatment outcomes [16,17]. Consequently, serious games have been shown to improve cognitive functioning and aid in the treatment of a variety of mental disorders, including depression and posttraumatic stress disorder [18].

Biofeedback is another technique used for anxiety management, which focuses on helping people gain control over their physiological functions. Since anxiety is linked to physical symptoms, like increased heart rate and respiratory issues, biofeedback can be an effective tool for detecting and treating anxiety [19]. Incorporating biofeedback therapy into games may increase the benefits of both techniques in terms of anxiety reduction [20,21].

In general, mobile apps and serious games have shown promise in self-managing anxiety; however, few apps targeting anxiety are available for Arabic speakers [22,23]. Culturally adapting the app design in terms of language, culture, and context and aligning it with the standards and values of the target population can improve user acceptance toward mental health apps [24]. Therefore, the goal of this study was to develop and evaluate the usability of a biofeedback-based app with serious games for young adults with anxiety in the UAE. The design of the app is customized to employ elements from the UAE culture.

## Methods

This study was conducted in two phases: (1) the development of a biofeedback-based app with serious games, and (2) the evaluation of the app’s usability with experts.

### Phase I: Development of a Biofeedback-Based App With Serious Games

#### Overview

**Figure 1:**
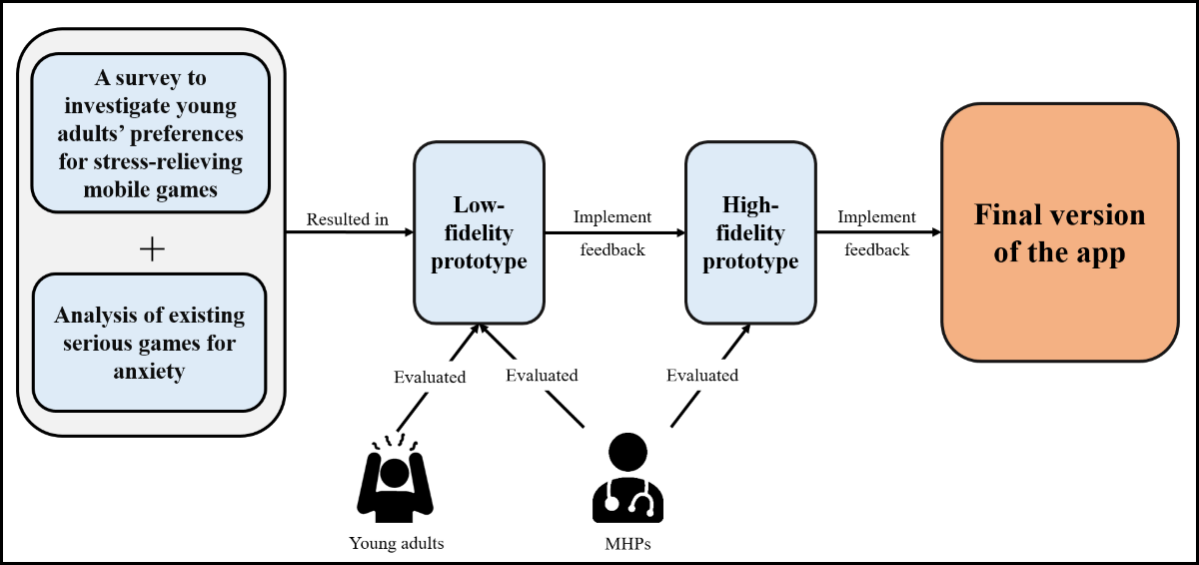
App development process. MHPs: mental health professionals.

In this phase, we used a user-centered design approach, which focuses on understanding the perspective of the target users. Therefore, the app was developed by combining data collected from young adults in the UAE and the results from analyzing existing serious games for anxiety as well as feedback from mental health professionals (MHPs). Figure 1 presents the development process of our app, which consists of 3 main parts.

#### Part 1: Investigation of Young Adults’ Game Preferences for Stress Relief

The goal of this part was to analyze the preferences of young adults (18-37 years old) toward mobile games in terms of functionality and design for stress and anxiety relief. Therefore, an online questionnaire, prepared using Google Forms, was sent via social media and mailing lists to university students and participation was voluntary. The questionnaire was available online for a period of 2 weeks, from November 19, 2020, to December 3, 2020. The estimated time for completion was 5 minutes and the participants’ answers were collected anonymously.

The online questionnaire consisted of 15 questions (7 multiple-choice questions, 3 yes/no questions, and 5 open questions). Four of the multiple-choice questions were multi-select. The questions were divided into three sections: demographics, stress-related, and game-related questions. For the student community, we used “stress” to replace “anxiety,” as participants might be unaware of the real meaning of anxiety and might refer to their anxiety as stress. Furthermore, high levels of stress can predict anxiety [25].

#### Part 2: Analysis of Existing Serious Games for Anxiety

The goal of this analysis was to observe two major issues: the characteristics of available serious games on mobile phones, and their overall design. Therefore, a systematic review was conducted in accordance with PRISMA (Preferred Reporting Items for Systematic Reviews and Meta-Analyses) guidelines [26]. A general search query composed of the terms “anxiety” and “game” was used and was automatically applied to the titles and descriptions of the games available in Google Play during March 2021. Each game from the search results was reviewed before deciding whether it should be included or excluded from the final selection. The following inclusion criteria were applied: (1) anxiety-related games in the Google Play store, (2) games that have a free version, and (3) games within the categories “Health & Fitness,” “Medical,” or “Brain Games.”

The categories were intended to highlight serious games that were primarily concerned with serious topics such as health and well-being, rather than games that were pure entertainment. Brain games, known as cognitive training, are specialized activities that mainly focus on enhancing attention and working memory [27], in turn having a role in relieving anxiety. To identify the final selection that would be examined, the following exclusion criteria were applied to the candidate games: (1) games that do not have ratings, and (2) games that could not be used after installation.

**Figure 2:**
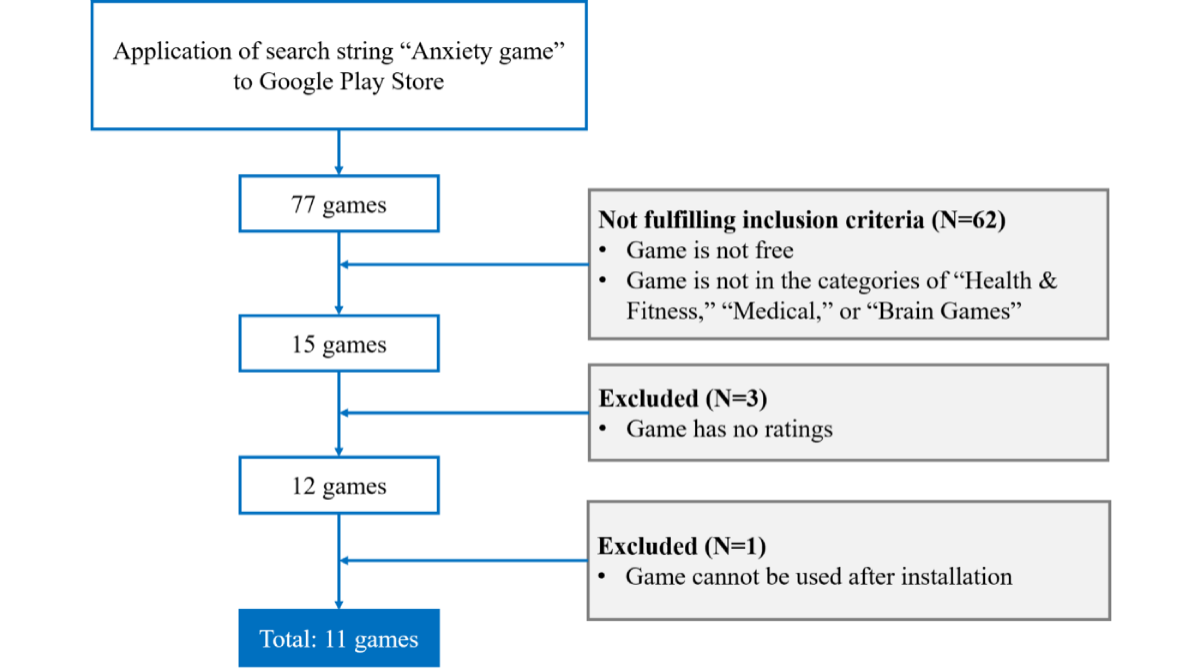
Selection process.

A total of 11 Android games were included in the final selection after the application of the aforementioned criteria (Figure 2). Each selected game was installed and examined on a Galaxy Note 9 (Android 10). A data extraction form was created in Microsoft Excel (Microsoft Corp). The games were classified into categories including puzzle, adventure, arcade, and simulation.

#### Part 3: Co-design With Mental Health Professionals

Two MHPs from the UAE were invited to participate in the co-design of the app. Both MHPs work as teaching assistants at UAEU College of Medicine, and are currently psychiatry and psychotherapy residents in Germany. They were chosen because they had previously worked with patients with anxiety in the UAE. Two evaluation cycles were conducted to refine the low-fidelity and high-fidelity prototypes. The evaluation was in the form of semistructured interviews with MHPs. The interviews were conducted online via Microsoft Teams and were digitally recorded to facilitate data analysis.

**Figure 3:**
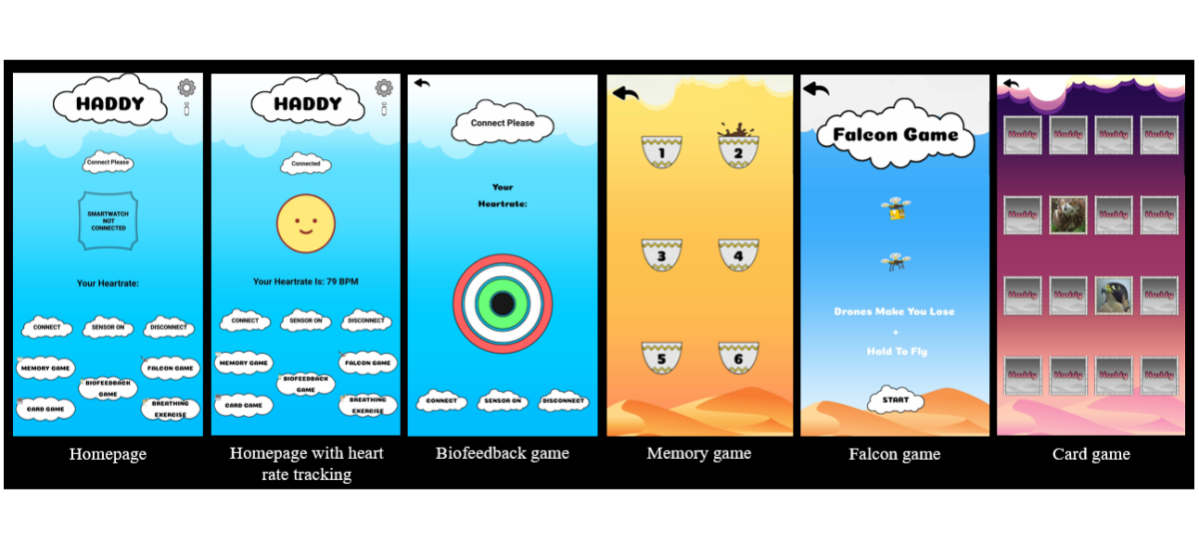
Screenshots of the low-fidelity prototype.

During the first cycle, the meeting started with a quick review of the project proposal, followed by a presentation of the low-fidelity prototype. Figure 3 presents pictures of the low-fidelity prototype screens presented to MHPs. The low-fidelity prototype was created based on the findings of Part 1 (Multimedia Appendix 1) and Part 2 (Multimedia Appendix 2) of Phase I. The main game, the biofeedback game, was developed in response to Part 1 findings indicating that young adults’ willingness to play games during stressful conditions increased if the game tracked heart rate and assisted with breathing exercises. Three key questions concerning the advantages, disadvantages, and potential modifications were asked after each proposed feature of the app. The findings were analyzed after the meeting to modify the prototype before cycle 2 with MHPs. Similarly, the low-fidelity prototype was also assessed by 6 university students aged between 18 and 25 years, who self-identified as having study anxiety. The interview was conducted and feedback on the prototype was solicited from the students. The prototype was well received by the students, with praise for the animation, types of games, and ease of use.

During the second cycle, MHPs evaluated the high-fidelity prototype, which was developed using Android Studio, and Tizen Studio for the smartwatch app. The Android Application Package (APK) for the app was emailed to the MHPs involved in cycle 1, along with a video that demonstrated the app’s primary features, before the second meeting. During the interview, 5 guiding questions were asked to cover topics including efficacy, preferences, design and graphics, engagement, and additional modifications. The following quotes demonstrate some of the feedback we received from MHPs after the second cycle:

- “Adding sound effects to the breathing exercise would have a positive impact on allowing the clients to relax”
- “The games would help with concentration and being present in the here and now. This will improve the overall mental state.”
- “The app will be useful for people with moderate anxiety”
- “Clients with severe anxiety would not be in a state of wanting to do such exercises”

### Phase II: Usability Evaluation

Data acquired from Phase I in this study resulted in an app named *Haddy*, which means “calm down” or “relax” in the Arabic Emirati dialect. In this phase, experts were involved in evaluating the usability of the final version of the app. In order to participate in this evaluation, an expert needed to have at least 3 years of experience in usability testing and software engineering. The research team used personal contacts to recruit 5 experts for this study. For the heuristic evaluation, 5 participants were considered a sufficient sample size [28].

Experts were required to download the app from the Google Play Store to evaluate the app. The evaluation was conducted using a questionnaire with a total of 30 items, 19 of which were based on Nielsen’s usability heuristics. Match between system and the real world, visibility of system status, memory, minimalist design, error prevention, consistency, user control, and flexibility are among the heuristics used in this evaluation. A 5-point Likert scale was used for the assessment (1=strongly disagree, 2=disagree, 3=neutral, 4=agree, 5=strongly agree). In addition, 9 items were included to score each feature of the app on a scale from 1 to 5, and 2 open questions were used to highlight the app’s advantages and problems related to the usability of the interface design.

### Ethics Approval

Ethical approval for the study was obtained from the relevant authorities at the United Arab Emirates University (ERS_2020_6156).

## Results

### Phase I: Development of a Biofeedback-Based Mobile App With Serious Games

**Figure 4:**
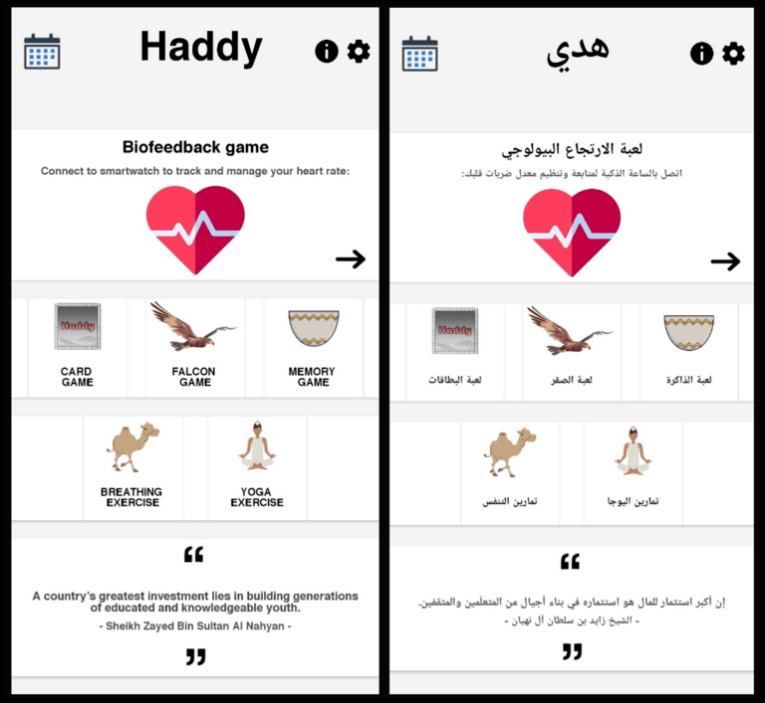
The final version of the app's homepage in English and Arabic.

The final version of the app (Figure 4) has the following key features: (1) biofeedback-based game, (2) card game, (3) falcon game, (4) memory game, (5) breathing exercise, (6) yoga videos, (7) positive messaging, and (8) mood tracking calendar. Since it is directed at people in the UAE, the app uses both Arabic and English languages.

**Figure 5:**
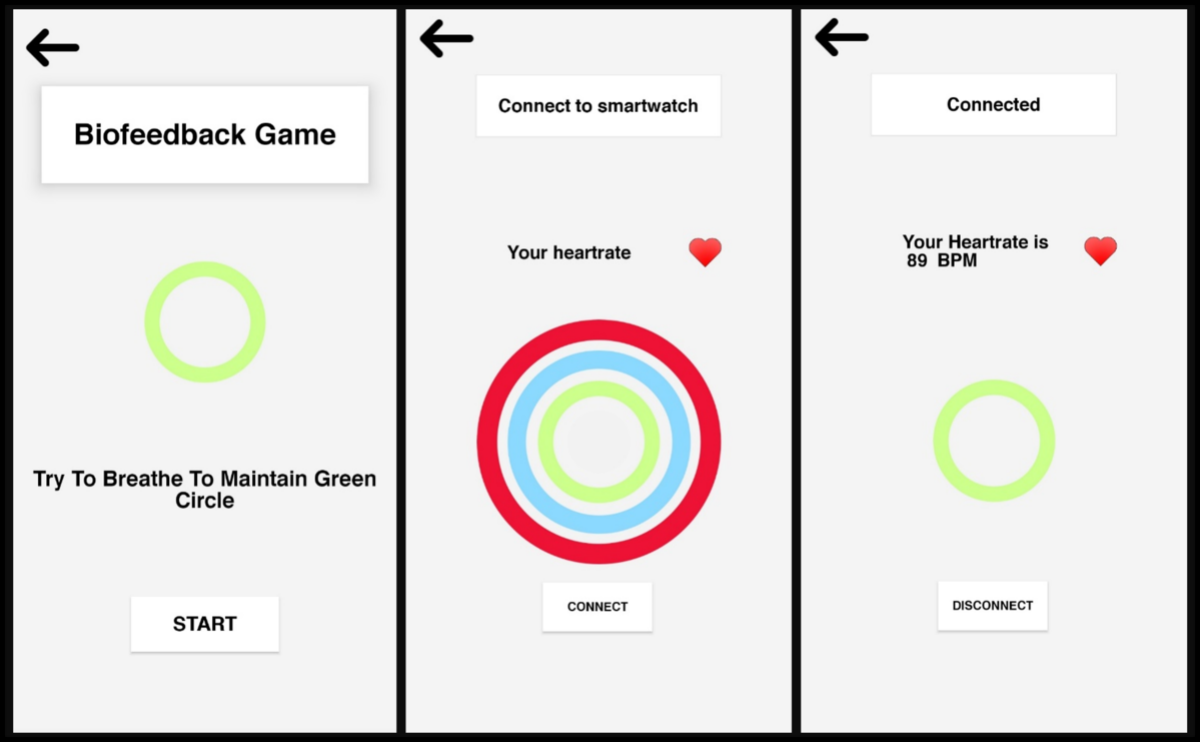
User interfaces for the biofeedback game.

The biofeedback game (Figure 5) was designed to help the player gain control over physical symptoms of anxiety. It requires the use of a smartwatch for heart rate tracking. To start the game, the player must first connect to the smartwatch by tapping the connect button. Prior to that, the user must put on the smartwatch and pair it with the phone via Bluetooth. After a few seconds of connection, the heart rate should appear. Heart rate is represented by colored circles: green (normal, at rest), red (high, anxious), and blue (low, dangerous). The normal heart rate falls between 60 and 100 beats per minute according to MHPs. When the color is not green, the player must relax by practicing activities such as breathing until the color goes back to green.

**Figure 6:**
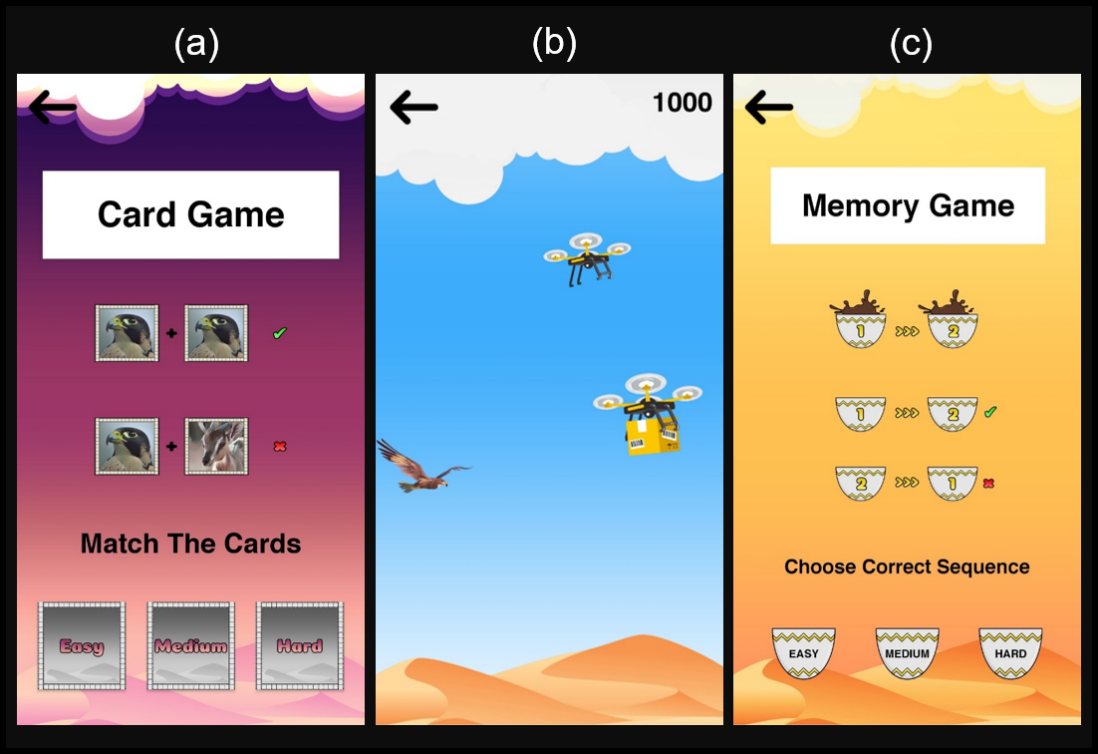
Mini-games including (A) the card game, (B) the falcon game, and (C) the memory game.

The card game (Figure 6A) is a classic mini-game with the goal of turning 2 matching cards at the same time. This game was designed to divert the user’s attention away from anxious thoughts. The game consists of three difficulty levels: easy (4×4 grid), medium (5×4 grid), and difficult (6×4 grid). The graphics on the cards portray animal species from the UAE, with a desert in the background. The game has no time or movement restrictions and it ends when all of the cards are acquired.

The falcon game (Figure 6B) is a side-view perspective arcade game used to divert someone’s attention away from negative thoughts and toward adjusting the falcon’s position. In this game, the falcon must fly through deadly drones to win. To keep the falcon flying, the player must keep pressing on the screen. Every 10 seconds, the score increases. If the falcon hits a drone, the game will end. If it does not, the game will continue, with the falcon’s speed increasing as the game proceeds. The highest score of the player will be saved.

The memory game (Figure 6C) is another game offered by the app, with a similar principle to the card game. A set of Arabic coffee cups is presented, and a sequence of pouring coffee into each cup begins one by one. The player must memorize and match the sequence in order to win the game. If the player chooses the incorrect order, the game will end. This game also presents three difficulty levels: easy (4-item sequence), medium (6-item sequence), and difficult (8-item sequence).

**Figure 7:**
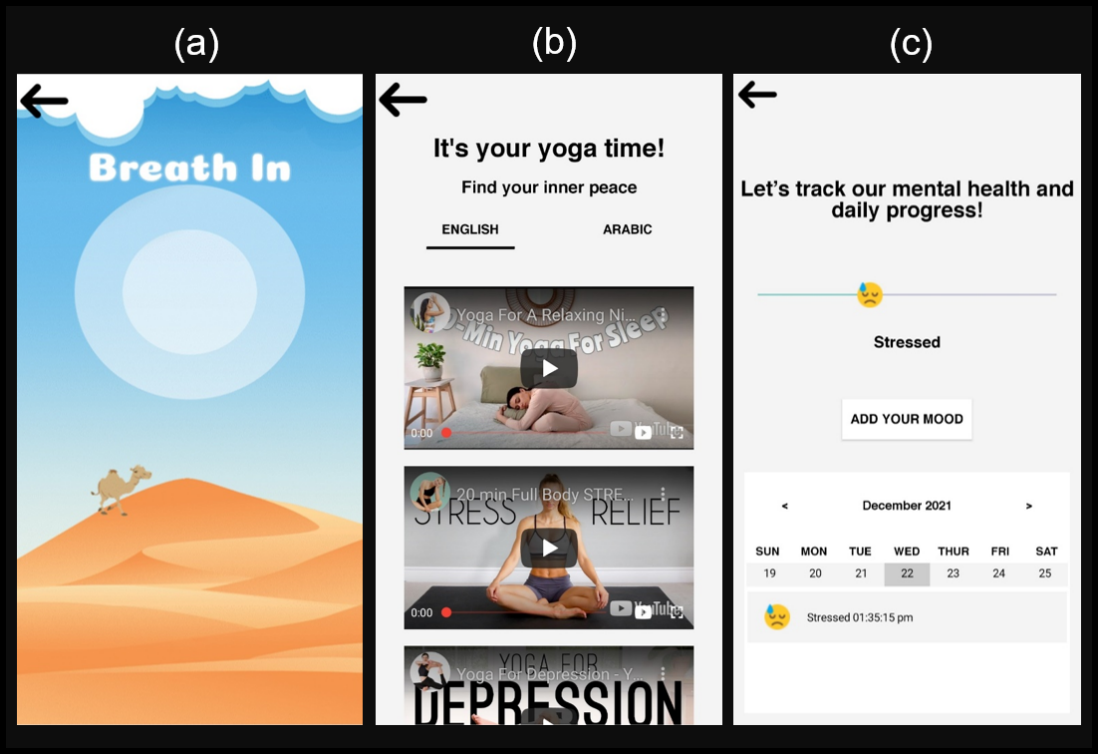
Other app functionalities include (A) a breathing exercise, (B) yoga videos, and (C) a mood tracking calendar.

The breathing exercise (Figure 7A) is a guided breathing exercise that instructs the user on when to inhale and exhale. Users are able to customize the duration of the exercise. They have four options: 2 minutes (which is the shortest), 5 minutes, 10 minutes, and 15 minutes (the longest). We added relaxing music to the breathing exercise based on feedback from the MHPs.

Yoga videos (Figure 7B) were included in both English and Arabic. There are a total of 12 yoga videos available, half in Arabic and half in English. The videos were obtained from YouTube channels; hence, they can only be viewed with internet access.

Positive messaging or quotes (Figure 4) were placed at the bottom of the homepage screen. Some of the quotes come from well-known Emirati public figures, such as the UAE’s beloved first president, Sheikh Zayed bin Sultan. When the user reopens the app, a new quote will appear.

A mood tracking calendar (Figure 7C) is a simple feature for people who wish to keep track of their mood on a daily basis. This can be useful when reflecting back on how one has been feeling over a period of time. Different moods can be observed if the user slides the progress bar. Users can define their current mood by selecting 1 of the 9 moods (tired, lonely, bored, stressed, I don’t know, calm, happy, excited, and wonderful) indicated with an icon and text. The user can add different moods in a single day by clicking the “Add mood” button. The moods will appear in chronological order in a weekly calendar.

### Phase II: Usability Evaluation

All 5 experts completed the usability evaluation questionnaire for the final version of *Haddy*. Table 1 shows the mean usability score from the experts’ point of view, with the consistency of the app scoring the highest. On the other hand, the ability to hide or display information, which is an aspect of minimalist design, was clearly low. In addition, both items of flexibility, including customization of functionalities and properties of interfaces, scored less than 4.

**Table 1:** Usability heuristics score (n=5).

Question			Mean (SD)^a^
**Match between system and the real world**			
	1. The app’s terminology is exposed in a familiar way to the user	4.6 (0.55)	
	2. The app icons and images are exposed in a familiar way to the user	4.4 (0.55)	
	3. The app’s screens are presented in the most logical way	4.0 (1.00)	
**Visibility of system status**			
	4. There is visual feedback that highlights the options that can be selected	3.6 (1.14)	
	5. There is no observable delay in the response time of the system	4.8 (0.45)	
	6. The app provides feedback to the user about its current status	4.4 (0.55)	
**Recognition rather than recall**			
	7. There is enough information displayed at each step in the tasks	4.4 (0.55)	
	8. The user can easily navigate back and forth without the need to remember each step	4.6 (0.55)	
**Esthetic and minimalist design**			
	9. The duplicated information is eliminated	4.8 (0.48)	
	10. It is possible to display or hide information (ie, expanding, collapsing lists)	2.4 (1.52)	
	11. The app design is minimalistic	4.4 (0.55)	
**Error prevention**			
	12. The app’s design minimizes the possibility of errors	4.6 (0.55)	
	13. The app warns the user of the error type in plain language	3.4 (1.52)	
**Consistency and standards**			
	14. The app remains consistent with elements that perform the same actions	4.8 (0.45)	
	15. The names of the options in the homepage are consistent in grammatical style and terminology	4.8 (0.45)	
**User control and freedom**			
	16. It is always possible to cancel the actioning of a task	4.2 (1.30)	
	17. It is easy for the user to undo and redo the actions	3.8 (1.30)	
**Flexibility and efficiency of use**			
	18. The user can change some properties of the interface	3.6 (0.89)	
	19. The app’s functionality can be customized by the user	3.2 (1.48)	
Rating scale of all items (total=95)			78.8 (9.58)

^a^All items were rated on a 5-point scale from 1=strongly disagree to 5=strongly agree.

According to the experts’ evaluation, the positive messaging and the card game were the most well-designed features of the app. Biofeedback, the falcon game, and the yoga videos, in contrast, had the lowest scores of all the features, but their scores remained over 4. Overall, the app design scored 4 out of 5. Table 2 shows the rest of the findings.

**Table 2:** Rating score for each feature in the app.^a^

Question	Participants						Mean (SD)
	P1	P2	P3	P4	P5		
Biofeedback game	4	4	3.5	5	4	4.1 (0.55)	
Card game	4	5	4.5	4	5	4.5 (0.50)	
Falcon game	4.5	5	4	4	3	4.1 (0.74)	
Memory game	4.5	5	4	4	4	4.3 (0.45)	
Breathing exercise	5	3	4.5	5	4	4.3 (0.84)	
Yoga videos	5	4	3.5	4	4	4.1 (0.55)	
Positive messaging	5	5	3.5	5	5	4.7 (0.67)	
Calendar mental status tracking	5	3	3.5	5	5	4.3 (0.97)	
Overall app design	4.5	4	4	4.5	3	4.0 (0.61)	

^a^All items were rated out of 5.

The last two questions in the usability questionnaire were designed to learn what experts liked and did not like about the app design. All the experts agreed that the app is simple and easy to use. One expert pointed out that the design suits the Arab region. On the other hand, experts identified some disadvantages, such as the amount of text displayed at once, improper error handling, and inconsistency with the display of icons. Along with the feedback, suggestions on how to improve the current version of the app were acquired and presented in Table 3.

**Table 3:** Suggestions provided by experts for improvement of the current version of the app.

Category	Suggestions
Minimization	Reduce the text on the instructions page of the breathing exercise, as the page is crowded.
Error handling	Display an error message to warn the user when the smartwatch is not detected in the biofeedback game.
Design	Improve the button design to make it clear where to click.
Consistency	Present the icon of the ‘mood tracking calendar’ in the same way as other features are displayed on the homepage. Improve the position of icons on the homepage.
Customization	Add “delete” and “edit” options in the mood tracking calendar. Allow for customization of the time duration in the breathing exercise. Provide mute/unmute options for the music in the breathing exercise. Allow full-screen viewing of yoga videos.

## Discussion

### Principal Findings

The goal of this study was to develop and test the usability of a biofeedback-based app with serious games for anxiety management for young adults in the UAE. According to the results of the questionnaire, games with heart rate tracking and breathing exercises have the potential to increase young adults’ interest in gaming as a stress-relieving method. This app was co-designed with MHPs in order to ensure the content’s integrity. The involvement of MHPs in the design process is thought to improve the efficacy of apps and to boost users’ trust in apps. A study has shown that the number of installs of anxiety apps that included MHPs was significantly higher than the number of installs of anxiety apps that did not include MHPs [29]. The target population for this app was young adults with mild to moderate anxiety symptoms, and not those with severe anxiety, since according to the MHPs involved in this study, those with severe anxiety were less likely to use this method of “self-help.” This is supported by findings by Christensen et al [30] that people with high anxiety symptoms were more likely to drop out of internet-based therapies. MHPs considered games as an effective technique to boost user concentration and shift attention away from anxious thoughts, resulting in better anxiety reduction outcomes. Additionally, audio was incorporated into the breathing exercise because sensory experiences such as music were emphasized for relaxation and reduction of anxiety [31,32].

Good usability ratings and positive feedback from experts may be in part due to the elicitation techniques used in Phase I, which included obtaining information from potential users, existing solutions, and MHPs. Experts recommended increasing customization, both for the interface and for particular features. Many users of anxiety and other mental health apps place more emphasis on customization [33]. Experts were generally satisfied with the app and found it easy to use, with only minor flaws that will be addressed in the next version. The findings of this study resulted in a future modification to facilitate the process of testing the app in young adults with anxiety.

### Recommendations

We believe that our study will provide developers and practitioners insight into potential and acceptable content and features, as well as different considerations while developing an app for young adults with anxiety in the UAE. Based on the findings, we recommend app developers to consider (1) the involvement of users and MHPs in the requirement elicitation process to improve usability; (2) reducing the amount of information presented in the user interface; (3) customization of the user interface as well as some elements of proposed features; and (4) handling all errors that may arise and ensuring the user is aware of them.

### Limitations

This study has a few limitations. The first limitation is that the usability of the high-fidelity prototype was evaluated by experts rather than young adults with anxiety; however, these results remain important, as the experts’ feedback will ensure that the app is suitable for use with the target population. Another limitation is that this study focused on the app’s interface conviviality, not the efficacy of the app in reducing anxiety symptoms, as this would require participant recruitment. We hope to overcome these limitations in future work by conducting a usability and efficacy evaluation with young adults with anxiety in the UAE. Finally, this app was initially developed for Android, but we intend to expand it to iOS in the future.

### Conclusions

This study has resulted in the development of a culturally sensitive biofeedback-based app with serious games to help young adults with anxiety in the UAE. *Haddy* was developed in collaboration with MHPs and tested by experts. The app has been well received by MHPs, with positive feedback about its ability to help with anxiety management. Experts who have evaluated the use of the app reported that it is simple and easy to use. Experts’ feedback will be included in the next version of the app. We intend to conduct a study to investigate the usability and impact of the future version of the app on a sample of young adults with anxiety in the UAE.

## Appendix

Multimedia Appendix 1 Questions and answers to the questionnaire that investigates young adults' preferences regarding games for stress relief.

Multimedia Appendix 2 Results of the analysis of Android mobile games for anxiety.

## Abbreviations

MHP: mental health professional
UAE: United Arab Emirates
