# Supplementary material for: A Biofeedback-Based Mobile App With Serious Games for Young Adults With Anxiety in the United Arab Emirates: Development and Usability Study
Source: JMIR Serious Games. 2022 Aug 2;10(3):e36936. doi: 10.2196/36936 (PMC9382548; doi:10.2196/36936)
Supplement: Multimedia Appendix 1 [file games_v10i3e36936_app1.docx]

| ID | Question | Answers | Frequency |
| --- | --- | --- | --- |
| **Demographic** | | | |
| Q1 | Age |  |  |
|  |  | <18 | 19 (9.2%) |
|  |  | 18-22 | 151 (73.3%) |
|  |  | 23-29 | 30 (15.6%) |
|  |  | ≥30 | 6 (2.9%) |
| Q2 | Gender |  |  |
|  |  | Male | 64 (31.1%) |
|  |  | Female | 142 (68.9%) |
| Q3 | Marital status |  |  |
|  |  | Single | 191 (92.7%) |
|  |  | Married | 12 (5.8%) |
|  |  | Other | 3 (1.5%) |
| **Stress-related questions** | | | |
| Q4 | How often do you feel stressed in a day? |  |  |
|  |  | Never | 20 (9.7%) |
|  |  | Once or twice | 111 (53.9%) |
|  |  | Very often | 75 (36.4%) |
| Q5 | What makes you feel stressed? |  |  |
|  |  | Work | 103 (50%) |
|  |  | Assignments/projects/exams | 172 (83.5%) |
|  |  | Family issues | 109 (52.9%) |
|  |  | Other | 6 (2.9) |
| Q6 | What do you think helps relieve stress? |  |  |
|  |  | Games | 107 (51.9%) |
|  |  | Food | 162 (78.6%) |
|  |  | Sleep | 120 (58.2%) |
|  |  | Sports | 81 (39.3%) |
|  |  | Watching TV | 85 (41.3%) |
|  |  | Other | 24 (11.7%) |
| **Games-related questions** | | | |
| Q7 | Will you be open to play mobile games to relax? |  |  |
|  |  | Yes | 97 (47.1%) |
|  |  | No | 46 (22.3%) |
|  |  | I don’t know | 63 (30.6%) |
| Q7.1 | If you answered no, please explain why: | Open answer |  |
| Q8 | Will you be willing to use a game that can help you do breathing exercises? |  |  |
|  |  | Yes | 119 (57.8%) |
|  |  | No | 25 (12.1%) |
|  |  | I don’t know | 62 (30.1%) |
| Q8.1 | If you answered no, please explain why: | Open answer |  |
| Q9 | Will you be willing to use a game that can track your heart rate to help you relax? |  |  |
|  |  | Yes | 123 (59.7%) |
|  |  | No | 26 (12.6%) |
|  |  | I don’t know | 57 (27.7%) |
| Q9.1 | If you answered no, please explain why: | Open answer |  |
| Q10 | What type of games would you like to play to relax? |  |  |
|  |  | Action games | 99 (48.1%) |
|  |  | Adventure games | 101 (49%) |
|  |  | Role-playing games | 40 (19.4%) |
|  |  | Simulation games | 39 (18.9%) |
|  |  | Strategy games | 48 (23.3%) |
|  |  | Sports games | 47 (22.8%) |
|  |  | Puzzle games | 101 (49%) |
|  |  | Idle games | 23 (11.25%) |
|  |  | Other: | 4 (1.9%) |
| Q11 | What kind of characters from UAE culture would you like to see in a game? |  |  |
|  |  | Camel | 84 (40.8%) |
|  |  | Falcon | 121 (58.7%) |
|  |  | Arabian Oryx | 57 (27.7%) |
|  |  | Sand cat | 59 (28.6%) |
|  |  | Sand gazelle | 74 (35.9%) |
|  |  | Other: | 3 (1.5%) |
| Q12 | What kind of features would you like to see in a mobile game to help you relax? | Open answer |  |
