# Supplementary material for: A Biofeedback-Based Mobile App With Serious Games for Young Adults With Anxiety in the United Arab Emirates: Development and Usability Study
Source: JMIR Serious Games. 2022 Aug 2;10(3):e36936. doi: 10.2196/36936 (PMC9382548; doi:10.2196/36936)
Supplement: Multimedia Appendix 2 [file games_v10i3e36936_app2.docx]

| **ID** | **Game name** | **Category** | **Rating** | **#Raters** | **Latest update** | **#Installs** |
| --- | --- | --- | --- | --- | --- | --- |
| A1 | DePuzzle - Anti Stress Brain Teaser Puzzle Game | Puzzle | 4.8 | 58 | 3-Mar-21 | 1,000+ |
| A2 | Music Zen - Relaxing Sounds | Other | 4.6 | 4822 | 5-Mar-21 | 100,000+ |
| A3 | eQuoo: Emotional Fitness Game | Adventure | 4.5 | 4,057 | 3-Jun-20 | 50,000+ |
| A4 | Antistress - relaxation toys | Simulation | 4.4 | 338,197 | 28-Feb-21 | 10,000,000+ |
| A5 | Stress Control Norbu: game, breathing, meditation | Puzzle | 4.4 | 1,361 | 1-Mar-21 | 100,000+ |
| A6 | Shadow's Edge - Resilience, Selfcare & Anti Stress | Adventure | 4.3 | 261 | 29-Dec-20 | 10,000+ |
| A7 | Upbeat Mind: Positivity Trainer | Puzzle | 4.3 | 16 | 3-Jan-20 | 1,000+ |
| A8 | Stress Relief Adult Color Book | Other | 3.9 | 1,318 | 10-Feb-21 | 100,000+ |
| A9 | Bubble wrap: Antistress, Anxiety Relief so Pop it | Arcade | 3.5 | 39 | 21-Jan-21 | 10,000+ |
| A10 | AWKWARD: Social Anxiety Endless Runner | Arcade | 3.2 | 106 | 14-Jul-20 | 5,000+ |
| A11 | Relax & Antistress Brain Games - Stress Relief App | Simulation | 3.0 | 60 | 1-Feb-21 | 10,000+ |
